# A Novel Glycolipid Biosurfactant Confers Grazing Resistance upon *Pantoea ananatis* BRT175 against the Social Amoeba *Dictyostelium discoideum*

**DOI:** 10.1128/mSphere.00075-15

**Published:** 2016-01-20

**Authors:** Derek D. N. Smith, Arvin Nickzad, Eric Déziel, John Stavrinides

**Affiliations:** a Department of Biology, University of Regina, Regina, Saskatchewan, Canada; b INRS-Institut Armand-Frappier, Laval, Québec, Canada; University of Wisconsin

**Keywords:** biosurfactants, *Pantoea*, *Dictyostelium*, opportunistic infections

## Abstract

*Pantoea* is a versatile genus of bacteria with both plant- and animal-pathogenic strains, some of which have been suggested to cause human infections. There is, however, limited knowledge on the potential determinants used for host association and pathogenesis in animal systems. In this study, we used the model host *Dictyostelium discoideum* to show that isolates of *Pantoea ananatis* exhibit differential grazing susceptibility, with some being resistant to grazing by the amoebae. We carried out a high-throughput genetic screen of one grazing-resistant isolate, *P. ananatis* BRT175, using the *D. discoideum* pathosystem to identify genes responsible for the resistance phenotype. Among the 26 candidate genes involved in grazing resistance, we identified *rhlA* and *rhlB*, which we show are involved in the biosynthesis of a biosurfactant that enables swarming motility in *P. ananatis* BRT175. Using liquid chromatography-mass spectrometry (LC-MS), the biosurfactant was shown to be a glycolipid with monohexose-C_10_-C_10_ as the primary congener. We show that this novel glycolipid biosurfactant is cytotoxic to the amoebae and is capable of compromising cellular integrity, leading to cell lysis. The production of this biosurfactant may be important for bacterial survival in the environment and could contribute to the establishment of opportunistic infections.

**IMPORTANCE** The genetic factors used for host interaction by the opportunistic human pathogen *Pantoea ananatis* are largely unknown. We identified two genes that are important for the production of a biosurfactant that confers grazing resistance against the social amoeba *Dictyostelium discoideum*. We show that the biosurfactant, which exhibits cytotoxicity toward the amoebae, is a glycolipid that incorporates a hexose rather than rhamnose. The production of this biosurfactant may confer a competitive advantage in the environment and could potentially contribute to the establishment of opportunistic infections.

## Abstract (precis)

The genetic factors used for host interaction by the opportunistic human pathogen *Pantoea ananatis* are largely unknown. We identified two genes that are important for the production of a biosurfactant that confers grazing resistance against the social amoeba *Dictyostelium discoideum*. We show that the biosurfactant, which exhibits cytotoxicity toward the amoebae, is a glycolipid that incorporates a hexose rather than rhamnose. The production of this biosurfactant may confer a competitive advantage in the environment and could potentially contribute to the establishment of opportunistic infections.

## INTRODUCTION

*Pantoea* is a genus of Gram-negative bacilli in the *Enterobacteriaceae* and a close relative of human-pathogenic genera *Klebsiella* and *Enterobacter. Pantoea* includes epiphytic and pathogenic members that have been identified throughout the environment, including clinical settings. Infections caused by members of *Pantoea* are known to occur primarily in immunocompromised individuals with preexisting conditions and can result in abscesses, pneumonia, or bacteremia (1–6); however, healthy individuals may be infected through penetrative injury by plant vegetation, which can result in cutaneous infections or develop into septic arthritis (6, 7). More concerning are the clinical cases of fatal bacteremia in neonates caused by contaminated parenteral nutrition as well as bacteremia in immunocompromised adults (1, 3–6, 8). Many members of *Pantoea*, such as *Pantoea ananatis*, while considered common environmental microbes, have been suggested to be opportunists with human-pathogenic potential (9, 10).

*P. ananatis* was originally isolated from pineapple as the causative agent of fruitlet brown rot (11). Many isolates have also been found to cause disease in a wide range of plant species but are also reported to colonize humans opportunistically (9). A study examining virulence potential showed that a *P. ananatis* pineapple isolate was especially virulent in an embryonated hen egg model compared to five clinical *Pantoea agglomerans* isolates (12). Similarly, quantitative growth assays have shown that closely related isolates of *P. ananatis* can vary greatly in their growth potential in plant and insect model hosts (13), suggesting the presence of specific genetic factors that mediate host association (14–16). This has been explored further in a comparative genomics analysis of eight *P. ananatis* genomes, which determined that isolates readily exchange genetic factors involved in host- and niche-specific colonization (17). Candidate disease factors were suggested to include a putative adhesin, multiple type VI secretion systems, and even type I fimbriae (17, 18). The presence of animal cell-specific type III secretion systems has also been noted in several strains, although their involvement in host association and specificity is still unclear (19, 20). There is currently limited functional information on these and other genetic factors in this species that may contribute to opportunism and human pathogenicity.

Model pathosystems are excellent tools for identifying candidate disease factors in pathogenic bacteria. *Dictyostelium discoideum* has been used as a model host for host-pathogen interactions due to its tractability, its similarities to mammalian cells in its cellular response to virulence factors, and its ability to phagocytose bacteria (21–25). Resistance to *D. discoideum* grazing can be used to identify isolates with possible virulence potential (26). *Pseudomonas aeruginosa* and *Burkholderia pseudomallei* mutants that are attenuated for virulence in the *D. discoideum* model are also attenuated in more complex model hosts like fruit flies and mice (22, 25). The *D. discoideum* pathosystem has been used to identify and assess the involvement of a variety of animal-specific virulence factors in host association, including the type VI secretion system, the type III secretion system, and various cytotoxins (25, 27–30). In this study, we used *D. discoideum* as a model host to screen for genetic factors that may enable *P. ananatis* to exploit animal hosts. One strain, *P. ananatis* BRT175, which was shown to have a grazing-resistant phenotype, was subjected to a genetic screen to identify the genetic determinants involved in resisting *D. discoideum* feeding. We show that two genes, *rhlA* and *rhlB*, are involved in producing a novel biosurfactant that is cytotoxic to *D. discoideum*.

## RESULTS

### Qualitative assay identifies grazing-resistant *P. ananatis* isolates.

**FIG 1:**
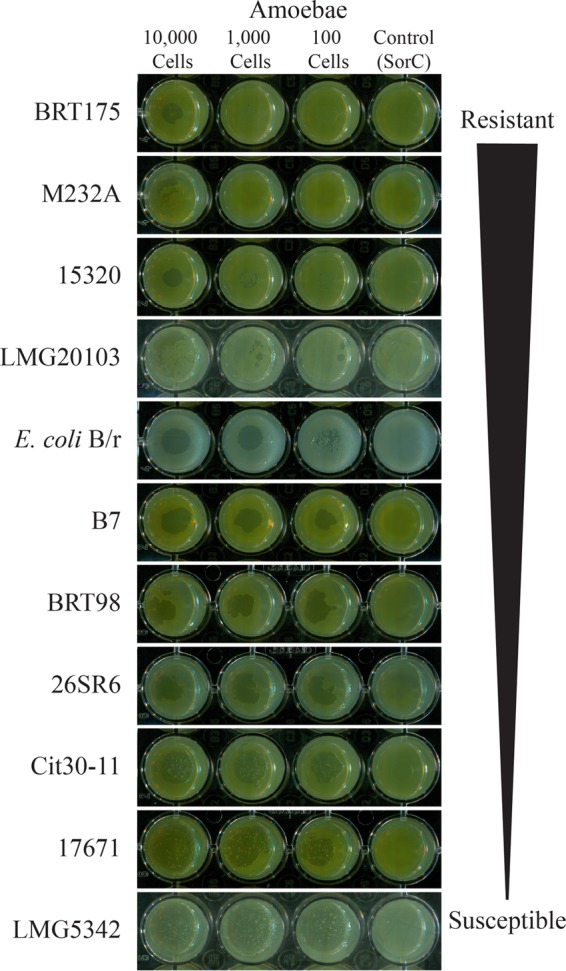
Qualitative assay of resistance of *P. ananatis* isolates to *D. discoideum* grazing. Ten thousand, 1,000, and 100 *Dictyostelium discoideum* AX2-214 cells were applied to dried bacterial lawns and monitored for 7 days for plaque formation and sporulation. *E. coli* B/r is used as the food source for *D. discoideum* and was included as a reference. The buffer SorC was used for the no-amoeba control.

The grazing resistance of 10 phylogenetically characterized *P. ananatis* isolates (13) was evaluated using a qualitative assay in which the plaque formation and sporulation on *Escherichia coli* B/r, the standard food source for *D. discoideum*, were used as a reference (Table 1). Grazing resistance was variable between the *P. ananatis* isolates and ranged from minimal plaque formation to full plaque formation and sporulation. *P. ananatis* BRT175 was the most resistant isolate, with minimal plaque formation and no sporulation when challenged with 10,000, 1,000, or 100 *D. discoideum* cells on modified SM (MSM) agar (Fig. 1). Similarly, *P. ananatis* M232A, LMG20103, and 15320 also showed a grazing-resistant phenotype with no sporulation. *P. ananatis* B7, BRT98, and 26SR6 showed an intermediate phenotype with large plaque formation occurring even when only 100 *D. discoideum* cells were applied. *P. ananatis* Cit30-11, LMG5342, and 17671 were completely susceptible to *D. discoideum* grazing, with large plaques forming and sporulation occurring in all treatments. Interestingly, the sister taxon isolate to *P. ananatis* BRT175, *P. ananatis* 17671, was one of the least grazing-resistant isolates, with plaque and spore formation being evident throughout the lawn. Grazing resistance and susceptibility did not appear correlated with phylogenetic relatedness within the *P. ananatis* species group (13), as isolates at both extremes of grazing resistance are closely related.

**TABLE 1:** Bacterial and eukaryotic strains used in this study

Species and strain	Selection	Source or reference
*Pantoea ananatis*	**	**
15320		Rice^a^
17671		Rice^a^
26SR6		Maize leaf^b^
B7	Rifampin	Maize^b^
BRT175	Rifampin	Strawberry^c^
BRT98	Rifampin	Strawberry^b^
Cit30-11	Rifampin	Navel orange leaf^b^
M232A		Maize^b^
LMG20103		Eucalyptus^d^
LMG5342		Human wound^d^
*Escherichia coli*		
HB101 (RK600)	Chloramphenicol	74
VPE42(pBSL118)	Kanamycin, ampicillin	37
B/r		DBS0305924 (75)
*Dictyostelium discoideum* AX2-214	Streptomycin, ampicillin	DBS0235534 (75)

^a^ International Collection of Microorganisms from Plants.

^b^ Steven Lindow, University of California, Berkeley.

^c^ Gwyn Beattie, Iowa State University.

^d^ Teresa Coutinho, University of Pretoria.

### Transposon mutagenesis identifies multiple genetic factors linked to grazing resistance.

To identify the genetic factors involved in grazing resistance, the grazing-resistant *P. ananatis* BRT175 was selected for mutagenesis and a high-throughput genetic screen was initiated for *D. discoideum* grazing susceptibility. A total of 3,789 mutants of *P. ananatis* BRT175 were screened, and 36 wells were identified as containing a grazing-susceptible mutant, as indicated by large plaque formation and/or sporulation. These 36 mutants were shown to represent 26 candidate genes that influence bacterial grazing resistance. Representative mutants from each of the 26 candidate genes were tested for growth deficiencies, with many of the mutants showing little or no defect in growth rate or growth density relative to the wild type (see Fig. S1 in the supplemental material). These 26 genes included quorum-sensing-related autoinducer synthase homologs of *phzI* and *eanI*, as well as purine and pyrimidine biosynthesis genes *guaB* and *pyrC*, *pyrD*, and *carB* (Table 2). Also identified was the *dsbB* gene, which is involved in a two-component system that forms disulfide bonds in periplasmic proteins (31–33), as well as the thioredoxin system component *trxB*, which plays an important role in replication during intracellular growth (34).

10.1128/mSphere.00075-15.1 Figure S1  Growth rate comparisons of mutants compared to wild-type *P. ananatis* BRT175. (A, B, and C) Growth curves are calculated from the mean OD_600_ readings of three independent experiments, and the shaded ribbon represents the 95% confidence interval. The *rhlA* mutant (28C8) and the *rhlB* mutant (32C5) exhibit growth rates similar to that of the wild type (BRT175). Download Figure S1, PDF file, 628 KB. Copyright © 2016 Smith et al. 2016 Smith et al. This content is distributed under the terms of the Creative Commons Attribution 4.0 International license.

**TABLE 2:** Locations of Tn*5* transposon gene insertions in grazing-susceptible mutants^d^

Grazing phenotype and locus^a^	Gene	Predicted function	Swarming	Mutant designation(s)
Grazing susceptible				
L585_12500	*ompR*	Transcriptional regulator	Y	28H12
L585_05285	*cpxA*	Histidine kinase	Y	35D8, 18A11
L585_21390	*phzR/luxR sdiA*	[Intergenic space-upstream] transcriptional regulator	Y	10E5
L585_04860	*ptsN*	PTS sugar transporter subunit IIA	Y	19E7
L585_22810	*nagC/nagD*	Transcriptional regulator (polar mutation of *nagC*)	Y	26A5
L585_16630	*dsbB*	Disulfide bond formation protein	Y	26H9
L585_21750	*serC*	3-Phosphoserine/ phosphohydroxythreonine aminotransferase	Y	28G12
L585_20040	*srmB*	RNA helicase	N	36D8
L585_10945	*yagG*	[Intergenic space-upstream] uncharacterized member of the GPH family of galactose-pentose-hexuronide transporters (symporter)	Y	31C1
L585_00065	*motA*	Flagellar motor protein	N	36E1
L585_00075	*flhD*	[Intergenic space-upstream] transcriptional regulator and flagellar apparatus operon	N	42A7, 42H1^b^
L585_10950	*eanI*	Acylhomoserine lactone synthase	Y	22A12
Enhanced grazing susceptible				
L585_10305		Hypothetical protein (*rrf2* domain, *badM*-like transcriptional regulator)	Y	16A5, 16A2,^b^ 16A1,^b^ 16H12^b^
L585_04880	*arcB*	[Intergenic space-downstream] aerobic respiration control sensor protein	Y	24A2, 24A5^c^
L585_16795	*prc*	Carboxy-terminal protease	Y	14G9
L585_21810	*trxB*	Thioredoxin reductase	Y	39D5
L585_03130	*rhlB*	Rhamnosyltransferase I, subunit B	Y	32C5, 24H12, 25E4, 40C2
L585_21385	*phzI*	Autoinducer synthase	Y	25E1, 26F4^b^
L585_03125	*rhlA*	Rhamnosyltransferase I, subunit A	N	28C8
L585_04515	*pyrB*	Aspartate carbamoyltransferase catalytic subunit	Y	36A4
L585_03535	*carB*	Carbamoyl phosphate synthase large subunit	Y	24G12
L585_19770	*guaB*	IMP dehydrogenase	Y	4H11
L585_21595	*pyrD*	Dihydroorotate dehydrogenase	Y	28H8
L585_04660	*pnp*	Polynucleotide phosphorylase/ polyadenylase	N	30D8
L585_15360	*prfC*	Peptide chain release factor 3	N	8F6
L585_21170	*pyrC*	Dihydroorotase	Y	35F10

^a^ Mutants exhibiting plaque formation were scored as grazing susceptible, whereas mutants in which 4 out of 8 test wells exhibited spore formation were classified as enhanced grazing susceptible. The NCBI accession number for the draft annotated whole-genome shotgun sequence is ASJH01000000.

^b^ Mutant not tested for swarming capability.

^c^ Mutant did not swarm.

^d^ Abbreviations: PTS, phosphotransferase; Y, yes; N, no.

**FIG 2:**
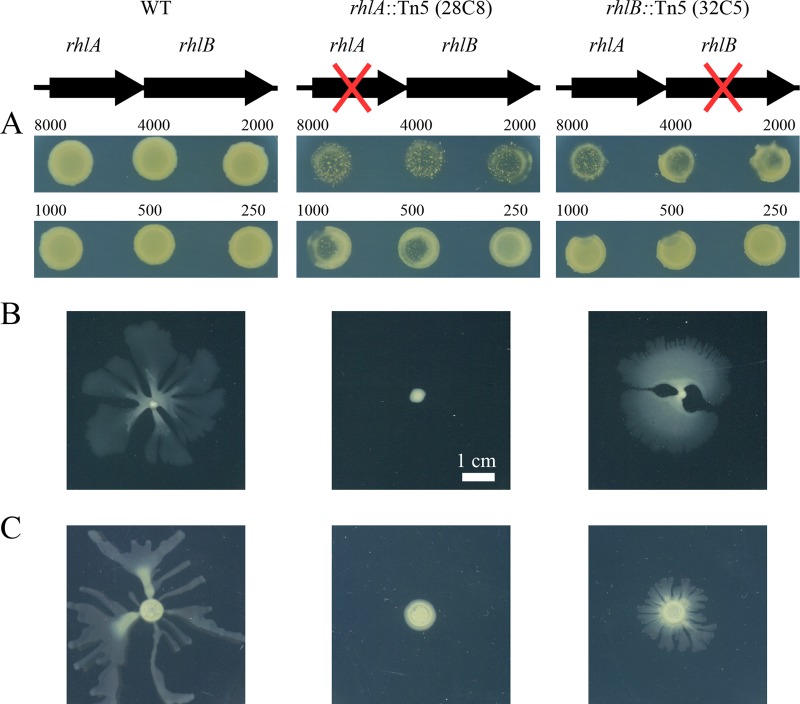
Grazing and swarming phenotypes of *Pantoea ananatis* BRT175 biosurfactant mutants. The predicted operon is shown at the top for each strain, with a red “X” indicating the disrupted gene. (A) Grazing of *P. ananatis* BRT175 at different cell densities (250 to 8,000 cells) of *D. discoideum* AX2-214 after a 7-day incubation. WT, wild type. (B) Swarming after a 24-h incubation on 0.5% modified SM agar. (C) Swarming after 48 h on 0.5% modified M9 agar.

The screen also identified *rhlA* and *rhlB* as being important for grazing resistance. The *rhlA* and *rhlB* genes, which were recovered once and four times, respectively (Table 2), are adjacent genes in the *P. ananatis* BRT175 genome. Homologs of these *rhlA* and *rhlB* genes encode enzymes responsible for the biosynthesis of rhamnolipids in *Pseudomonas aeruginosa* and a few *Burkholderia* species (35). The transposon insertion in the *rhlA*::Tn*5* mutant (mutant 28C8) occurred near nucleotide 385 of the 837-bp open reading frame, which would induce a polar mutation if the genes are coregulated and in a putative operon (36, 37). The transposon insertion in the *rhlB*::Tn*5* mutant (mutant 32C5) occurred near nucleotide 531 of the 1,173-bp open reading frame. Both *rhl* mutants had severe impairment in grazing resistance, with amoebal sporulation being evident after 7 days of grazing compared to no sporulation and minimal plaque formation on wild-type *P. ananatis* BRT175. The *rhlA*::Tn*5* mutant was even more susceptible, showing complete grazing susceptibility with as few as 500 amoebae being sufficient for sporulation, whereas 2,000 or more amoebae were necessary for sporulation on the *rhlB*::Tn*5* mutant (Fig. 2A).

### *rhlA* is involved in biosynthesis of a glycolipid biosurfactant.

**FIG 3:**
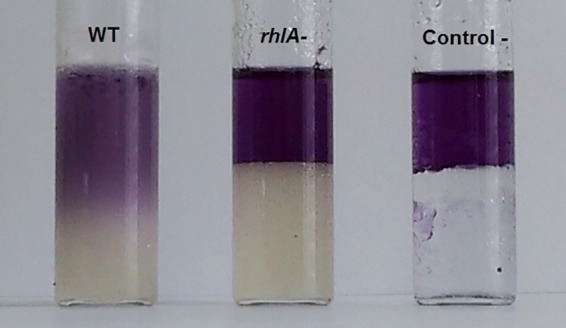
Emulsification activity of culture extracts of *P. ananatis* BRT175 strains against *n*-hexadecane. The wild-type (WT) extract exhibits emulsifying activity, whereas the extract from the *rhlA*::Tn*5* (mutant 28C8) culture lacks any emulsifying activity, with the height of the hydrophobic phase being identical to the negative control (reagent-grade water).

To establish the involvement of a biosurfactant, we measured and compared surface tension and emulsification activities of culture extracts from *P. ananatis* BRT175 and the *rhlA*::Tn*5* mutant. Wild-type culture extracts were found to reduce the surface tension of water from 72 to 40 mN/m, whereas the *rhlA*::Tn*5* culture extracts could reduce the water surface tension only to 62 mN/m. Accordingly, the mutant extracts had almost no emulsification activity compared to the wild type (Fig. 3). These results suggested the production of an extracellular biosurfactant and the involvement of the *rhlA* product in its synthesis.

**FIG 4:**
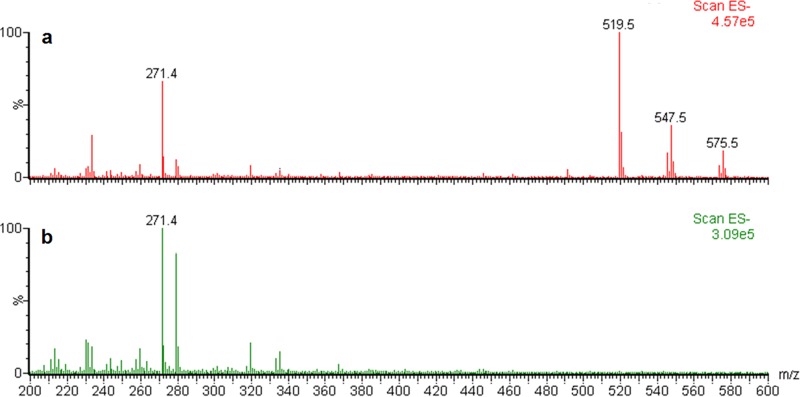
Triple-quadrupole ESI-MS spectra of *P. ananatis* BRT175 culture extracts obtained in negative ionization mode. Pseudomolecular ions [M-H]^−^ are observed at *m/z* 519, 547, and 575 for the wild-type strain (a), which are absent in culture extracts of the *rhlA*::Tn*5* strain (mutant 28C8) (b). The *y* axis shows relative abundance considering the highest ion as 100%; the *x* axis shows *m/z* for each ion. The pseudomolecular ion at *m/z* 271 corresponds to 16-hydroxyhexadecanoic acid, which was added as an internal standard.

**FIG 5:**
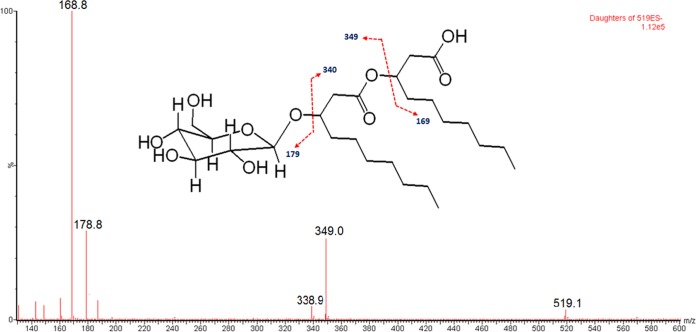
Daughter ions produced upon fragmentation of the *m/z* 519 pseudomolecular ion, using MS/MS. The proposed structure and fragmentation pattern are illustrated.

To determine if the biosurfactant was a rhamnolipid, we analyzed the extracts from the wild type and the *rhlA*::Tn*5* cultures using liquid chromatography-mass spectrometry (LC-MS) protocols developed for rhamnolipids. Wild-type extracts had three peaks with *m/z* values of 519, 547, and 575, which were absent from the *rhlA*::Tn*5* culture extracts (Fig. 4), but none of these corresponded to any of the approximately 60 different known rhamnolipid congeners (38). To investigate this unexpected result, we used tandem mass spectrometry (MS/MS) fragmentation to further analyze the *m/z* 519 pseudomolecular ion, the most abundant molecule requiring *rhlA* for its biosynthesis. This biosurfactant was determined to be a monohexose-C_10_-C_10_, although the exact nature of the sugar moiety is still undetermined (Fig. 5). The two other identified masses (*m/z* 547 and *m/z* 575) appear to correspond to congeners containing hydroxydodecanoic (C_12_) and hydroxytetradecanoic (C_14_) acid moieties as side chain fatty acids. Further investigation will be required to fully identify the exact nature of these new biosurfactants.

### *rhlA* but not *rhlB* is essential for full swarming motility.

**FIG 6:**
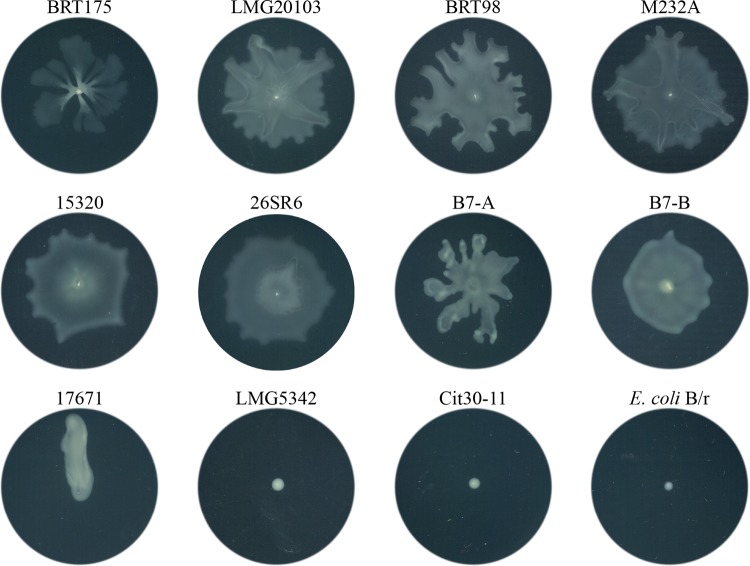
Swarming motility patterns of wild-type *P. ananatis* strains and *E. coli* B/r. All images represent the motility patterns observed after incubation for 24 h at 21°C following a single point inoculation on 0.5% modified SM agar. B7-A and B7-B represent two different swarming phenotypes of *P. ananatis* B7.

Because rhamnolipids can aid in swarming motility in some species (39–41), we compared the swarming proficiency of the *rhl* mutants relative to that of the wild type. Wild-type *P. ananatis* BRT175 exhibits a characteristic dendritic pattern on 0.5% modified SM agar (Fig. 2B and C), whereas the *rhlA*::Tn*5* mutant is deficient in swarming on 0.5% modified SM agar and 0.5% modified M9 agar (Fig. 2B and C). The *rhlB*::Tn*5* mutant was still capable of swarming on both medium types (Fig. 2B and C). The three other *rhlB*::Tn*5* mutants (25E4, 24H12, and 40C2), each of which appeared to have a unique transposon insertion site, also retained swarming activity on 0.5% modified SM agar. To determine whether swarming motility was an indicator for grazing resistance, all isolates in the *P. ananatis* group were tested for swarming proficiency. Grazing-susceptible *P. ananatis* Cit30-11 and LMG5342 and *E. coli* B/r showed no motility (Fig. 6), whereas *P. ananatis* 17671, a grazing-susceptible isolate, had a different motility phenotype, as it appeared to move mostly linearly from the point of inoculation. *P. ananatis* 17671 did not form dendrites, nor did it form what is commonly referred to as a “featureless mat” (42), which is one of the motility patterns formed by some swarming bacteria. *P. ananatis* LMG20103, M232A, B7, 15320, BRT98, and 26SR6, which represent both grazing-resistant and intermediate strains, formed primarily a “featureless mat” phenotype with some dendrite-like protrusions visible in B7, LMG20103, M232A, and BRT98 trials (Fig. 6).

### The biosurfactant produced by *P. ananatis* BRT175 is cytotoxic to *D. discoideum.*

**FIG 7:**
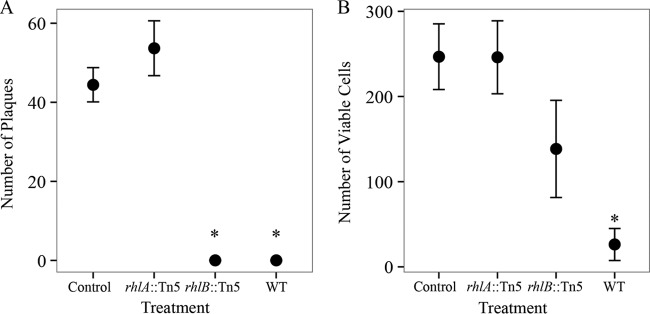
Mean plaque and cell viability counts after exposure to conditioned culture medium. The figure was generated in R using ggplot2 (72, 73). (A) Plaque-forming units were enumerated after 25 min of room-temperature exposure to medium conditioned with either wild-type *P. ananatis* BRT175, the *rhlA*::Tn*5* strain (mutant 28C8), or the *rhlB*::Tn*5* strain (mutant 32C5). Each data point represents the mean from three independent experiments with the standard error reported. The asterisk denotes statistical significance using Dunn’s test (*P* = 0.0305). (B) *D. discoideum* AX2-214 cells were enumerated after exposure to medium conditioned with either wild-type *P. ananatis* BRT175, the *rhlA*::Tn*5* strain (mutant 28C8), or the *rhlB*::Tn*5* strain (mutant 32C5) for 20 min at room temperature. Each data point represents the mean from three independent experiments with the standard error reported. The asterisk denotes statistical significance using Dunn’s test (*P* = 0.0064).

To evaluate whether the new biosurfactant produced by *P. ananatis* BRT175 has a cytotoxic or inhibitory effect at concentrations produced within culture media, *D. discoideum* cells were exposed to cell-free culture medium conditioned with either wild-type *P. ananatis* BRT175, the *rhlA*::Tn*5* mutant, or the *rhlB*::Tn*5* mutant (Kruskal-Wallis test, significant *P* = 0.02). In three independent plaque assay experiments, no plaques formed on plates seeded with amoebae that had been exposed to wild-type-conditioned culture medium (*P* = 0.0305) (Fig. 7A). Amoebae exposed to *rhlA*::Tn*5* strain-conditioned medium formed plaques similar to the number seen in the control (*P* = 0.2728). Interestingly, the *rhlB*::Tn*5* strain-conditioned medium also did not allow for plaque formation (*P* = 0.0305). Amoeba cell viability assays using trypan blue staining were consistent with these results (Kruskal-Wallis test, *P* = 0.04), with exposure to the wild-type-conditioned culture medium leaving mostly cellular debris (*P* = 0.0064) (Fig. 7B). Cells exposed to the *rhlB*::Tn*5* strain-conditioned medium were still largely intact, but many cells showed trypan blue uptake, indicating loss of membrane integrity (*P* = 0.1065); there was also no significant difference between the *rhlA*::Tn*5* strain-conditioned medium and the control (*P* = 0.4549).

### *rhlA* and *rhlB* are distributed among isolates of *P. ananatis.*

**FIG 8:**
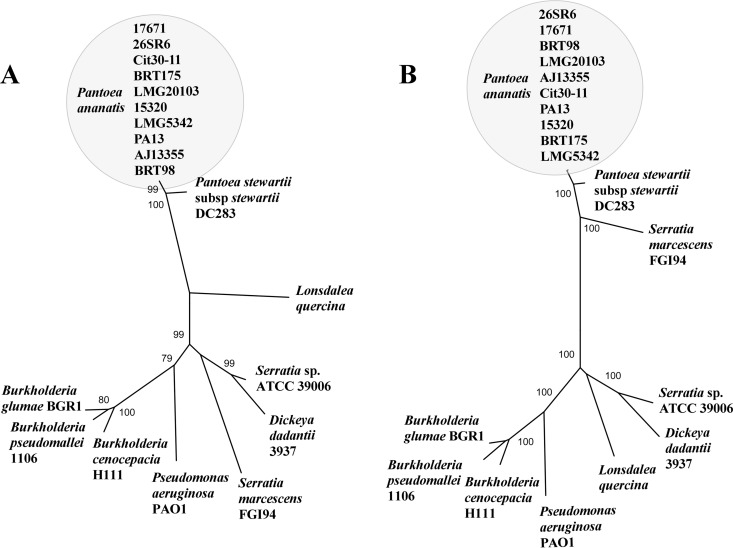
Phylogenetic trees of RhlA and RhlB homologs. Radial (unrooted) neighbor-joining phylogenies of representative RhlA (A) and RhlB (B) homologs, constructed using the Jones-Taylor-Thornton amino acid model, with complete gap deletion. Only bootstrap values greater than 70% are shown. In addition to the *P. ananatis* strains used in this study, other representative taxa included *Burkholderia cenocepacia* H111 (CDN64069), *Burkholderia glumae* BGR1 (ACR31008), *Burkholderia pseudomallei* 1106a (ABN94800), *Dickeya dadantii* 3937 (YP_003882762), *Lonsdalea quercina* (WP_026742016), *Pantoea stewartii* subsp. *stewartii* DC283 (EHU02365), *Pseudomonas aeruginosa* PAO1 (NP_252169), and *Serratia marcescens* FGI94 (AGB82844).

A survey of the distribution of the *P. ananatis* BRT175 *rhlA* and *rhlB* genes using BLAST identified the closest homologs in several other sequenced isolates of *P. ananatis*, including *P. ananatis* LMG20103 (43), LMG5342 (44), AJ13355, and PA13, as well as in unpublished draft genomes of *P. ananatis* 15320, BRT98, 26SR6, Cit30-11, and 17671. Homologs were also identified in *Pantoea stewartii* subsp. *stewartii* DC283, which is a sister species to *P. ananatis* (13). Independent genealogies of the two genes constructed using representative sequences from a variety of species show partial congruence of the major clades, with monophyly of the *P. ananatis*-*P. stewartii* homologs; however, the most recent common ancestor of the *P. ananatis rhlA* gene is shared with *Lonsdalea quercina*, whereas the *P. ananatis rhlB* gene is more closely related to the *Serratia marcescens* homolog (Fig. 8). In the *Serratia* and *Dickeya* lineages, the *rhlA* and *rhlB* genes are not adjacent to each other as in the *P. ananatis* isolates.

## DISCUSSION

This work explored the genetic factors responsible for grazing resistance and/or virulence of *P. ananatis* in a *D. discoideum* pathosystem. The qualitative grazing assay identified four out of 10 isolates as having a resistant phenotype (limited plaque formation), three as having intermediate phenotypes (plaque formation but no sporulation), and three as having a susceptible phenotype (plaque and spore formation) (Fig. 1). Sister isolates *P. ananatis* BRT175 and *P. ananatis* 17671 showed phenotypes on the two extremes of the grazing resistance spectrum, supporting the presence of specific genetic factors that contribute to this phenotype. It has been shown that there are differences in the flexible genomic complement of *P. ananatis* isolates, such that the species appears to have an open genome (17), which facilitates the introduction of substantial genetic variability and the acquisition of isolate-specific virulence traits. In the case of grazing resistance, the method by which such factors confer the grazing-resistant phenotype may be through one of several mechanisms. The plaque formation with no sporulation, for example, may suggest that the bacteria interfere with the *D. discoideum* life cycle, as shown with *Salmonella enterica* subsp. Typhimurium (45). *S. enterica* inhibits the *D. discoideum* starvation response through the type III secretion system (45), thereby preventing sporulation. We were unable to identify any type III secretion system in *P. ananatis* BRT175 that might function analogously (46). However, isolates within the *P. ananatis* group may use other secretion systems, which are known to be responsible for producing cytotoxic factors by some bacteria (18, 47). It is also possible that bacteria produce antifeeding factors, such that the amoebae starve to death. *Serratia marcescens* produces serrawettin W2, a surfactant that causes *Caenorhabditis elegans* to avoid feeding on the bacteria (48). Another mechanism to explain the grazing phenotype is the direct killing of the predatory amoebae. A wide variety of toxins and virulence factors are activated in a density-dependent manner through quorum sensing regulation in a number of pathogenic bacteria (49–51), which may directly target the amoebae. Several of the 26 candidate loci implicated in grazing resistance were genes involved in biosynthetic pathways that had been shown previously to be involved in virulence in humans and animal model systems (Table 2).

Of the candidate genes identified, the rhamnolipid biosynthesis genes *rhlA* and *rhlB* were of particular interest due to their involvement in the production of rhamnolipids and their importance in the virulence of the opportunistic human pathogens *P. aeruginosa* and *Burkholderia* spp. (52–54). Typically, *rhlA* and *rhlB* are in an operon (55, 56), and this may be the case for *P. ananatis* BRT175 based on the collinearity and close proximity of the two coding regions in the genome sequence (46). In other systems, these genes direct the production of rhamnolipids, which act as wetting agents that enable swarming motility but which also have cytotoxic properties (40, 53). RhlA is necessary for the synthesis of 3-(3-hydroxyalkanoyloxy)alkanoic acids (HAA) (40). RhlB is the rhamnosyltransferase that transfers a dTDP-l-rhamnose to HAA to form a monorhamnolipid, while RhlC can transfer an additional rhamnose sugar to create a dirhamnolipid (40, 55, 57). Interestingly, these glycolipidic biosurfactants have been reported only anecdotally in cultures of *Enterobacteriaceae* (38). In *P. ananatis* BRT175, the products of *rhlA* and *rhlB* are responsible for the biosynthesis of a novel, hexose-based surfactant. The lipid moieties primarily consist of hydroxydecanoic (C_10_) acid, much like the rhamnolipids produced by *P. aeruginosa* (58), but other congeners that likely consisted of hydroxydodecanoic (C_12_) or hydroxytetradecanoic (C_14_) acid were detected. This glycolipid also exhibits emulsification and surface tension reduction properties, which are known characteristics of rhamnolipid biosurfactants (38).

The culture medium conditioned by the growth of *P. ananatis* BRT175 was cytotoxic based on our observations in a plaque assay and trypan blue staining of exposed cells. The cytotoxicity of the wild-type-conditioned medium is consistent with previous work in *P. aeruginosa* that showed rapid lysis of *D. discoideum* cells (30) and polymorphonuclear leukocytes (53) after exposure to rhamnolipid extracts. While we did not observe plaque formation after exposure to *rhlB*::Tn*5* strain supernatant, trypan blue staining revealed compromised cells, suggesting the retention of some level of cytotoxicity likely due to the production of the fatty acid component of the biosurfactant by RhlA (Fig. 2A). In *P. aeruginosa*, the HAA precursor still possesses some surfactant properties (39, 40), which also explains the capacity of the *rhlB*::Tn*5* strain to still swarm, albeit differentially from wild type (Fig. 2B and C). Rhamnolipid-free and HAA-dependent *P. aeruginosa* swarming can lead to a reduced swarming zone (40) and altered motility patterns (39). Another study examining the influence of rhamnolipids and HAA on swarming motility patterns found that HAA can inhibit dendritic tendril formation and can cause repulsion of cells away from concentrations of HAA (59).

Our analysis of the distribution of the *rhlA* and *rhlB* homologs revealed that these two genes are common to all *P. ananatis* strains analyzed. The qualitative differences in the grazing resistance phenotypes between *P. ananatis* BRT175, *P. ananatis* BRT98, *P. ananatis* LMG20103, *P. ananatis* LMG5342, *P. ananatis* Cit30-11, *P. ananatis* 26SR6, *P. ananatis* 17671, and *P. ananatis* 15320 are not attributable solely to the presence of *rhlA-rhlB* genes, as all 8 genomes carry the genes (Fig. 8). This suggests that differences in regulation, other unidentified genetic factors, or combinations thereof are involved in the *P. ananatis*-*D. discoideum* interaction. Nonetheless, the persistence of these genes in the *P. ananatis* lineage suggests that they play an important role in the biology of this species. Furthermore, the presence of these genes in the sister species *P. stewartii* could suggest acquisition by the *P. ananatis-P. stewartii* common ancestor, although additional *P. stewartii* isolates will need to be surveyed to test this hypothesis. The origin of acquisition is not entirely clear, particularly given that the closest homolog of the *P. ananatis-P. stewartii rhlA* gene is that from the enteric bacterium *L. quercina*, whereas the *P. ananatis-P. stewartii rhlB* gene is closest to the homolog from a different enteric bacterium, *S. marcescens* (Fig. 8). Because the homologs are not adjacent in the *Serratia* genome, this could indicate that *P. ananatis rhlA* and *rhlB* have come from different sources. Alternatively, the *Pantoea* homologs may have been transferred to some of the other enteric organisms individually, although there are insufficient representative sequences from this group to determine whether this is the case.

The evolutionary arms race between microorganisms competing for the same environmental niche is thought to be an evolutionary driver for opportunistic pathogens (60–62). This predatory relationship of environmental amoebae with bacteria, for example, may select for traits in prey that confer resistance to phagocytosis but which have the potential for exaptation as virulence factors that function against the cells of the innate immune system (30, 53, 61, 63). Such determinants may enable those same isolates to exploit additional hosts, effectively expanding their host range to include animals and humans. Unraveling the key genetic factors that enable strains of *Pantoea* spp. to colonize different hosts will provide important insight into the pathogenic capabilities of this highly versatile group.

## MATERIALS AND METHODS

### Cell growth and culturing.

Overnight cultures of *Pantoea* and *Escherichia coli* were grown at 30°C and 37°C, respectively, in Miller LB broth (BD, Franklin Lakes, NJ) with shaking at 220 rpm under appropriate antibiotic selection and conditions (Table 1) (50 µg/ml kanamycin, 150 µg/ml ampicillin, 50 µg/ml rifampin, 38 µg/ml chloramphenicol). *Dictyostelium discoideum* AX2-214 cultures were incubated at 21°C and maintained in petri dishes containing 10 ml HLF1 (Formedium, Hunstanton, United Kingdom) HL5 medium with vitamins and microelements, supplemented with 13.5 g/liter glucose, 300 µg/ml streptomycin, and 150 µg/ml ampicillin. Cells were passaged every 4 to 6 days following a 100-fold dilution, and fresh stocks were prepared axenically from spores after 3 to 4 weeks of passaging (64). Spores were harvested from SM/5 plates (2 g/liter glucose, 2 g/liter Bacto peptone [BD], 0.2 g/liter yeast extract [BD], 0.2 g/liter MgSO_4_⋅7H_2_O, 1.9 g/liter KH_2_PO_4_, 1.0 g/liter K_2_HPO_4_, 1.5% agar [BD], pH 6.5) with *E. coli* B/r as a food source (64). Cultures for downstream assays were prepared via a 100- to 200-fold dilution of confluent cells in Erlenmeyer flasks containing HL5 at no more than 20% of the maximum volume. The cells were incubated at room temperature and shaken at 180 rpm. Cells were harvested during log-phase growth (typically 3 × 10^6^ to 6 × 10^6^ cells/ml) by centrifuging 15 to 50 ml of cell culture three times at 500 × *g* for 5 min and resuspending the pellet each time in Sorensen’s buffer with calcium (SorC) (2 g/liter KH_2_HPO_4_, 0.29 g/liter Na_2_HPO_4_, 50 µM CaCl_2_, pH 6.0). The final pellet was resuspended in 1 ml of SorC. Cells were enumerated in a hemocytometer and diluted to appropriate concentrations for downstream applications.

### Qualitative screen for grazing-resistant isolates.

A collection of *Pantoea* isolates (13) was evaluated for grazing resistance using a modified qualitative *D. discoideum* assay (26). Cells from an overnight *Pantoea* culture were centrifuged at 10,000 × *g* for 5 min and resuspended in an equal volume of SorC twice. To ensure consistency of inoculations across qualitative grazing plates, bacterial cell densities were standardized to a final optical density at 600 nm (OD_600_) of 1.0 in SorC by taking absorbance readings from 200-µl samples using a BioTek Epoch microplate spectrophotometer (BioTek, Winooski, VT) in 96-well plates (Greiner Bio-One, model 655180; Monroe, NC). Fifty microliters of each bacterial suspension was spotted and allowed to dry onto 24-well plates containing 1.5 ml modified SM agar per well (65) (10 g/liter glucose, 10 g/liter Bacto peptone [BD], 1 g/liter yeast extract [BD], 1 g/liter MgSO_4_⋅7H_2_O, 1.9 g/liter KH_2_PO_4_, 0.6 g/liter K_2_HPO_4_, 1.5% agar [BD], pH 6.5). Suspensions of *D. discoideum* were pipetted in 5-µl aliquots onto the center of each well at 10,000, 1,000, and 100 total cells. *Escherichia coli* B/r, which is used as the food source for *D. discoideum*, served as a control. Isolates were identified as grazing resistant if plaque formation was reduced or absent and sporulation did not occur after exposure to 10,000 *D. discoideum* cells after 7 days of incubation at 21°C (in darkness). The experiment consisted of two replicates, and experiments were repeated twice with similar results.

### Genetic screening and mutant characterization.

Mutagenesis was carried out using a triparental mating approach, whereby the RK600 helper plasmid was used to introduce the pBSL118 plasmid, carrying a mini-Tn*5* transposon (kanamycin resistance), into *P. ananatis* BRT175 (rifampin resistance) (Table 1). Aliquots of each overnight culture (1 ml) were pelleted and resuspended in 100 µl LB and mixed in a 1:1:1 ratio. This triparental mixture (100 µl) was spotted on LB agar plates and incubated overnight at 30°C. A portion of the bacterial lawn was then scraped from the plate with the edge of a sterile spreader and spread on an LB plate containing rifampin and kanamycin. Following incubation for 24 to 48 h at 30°C, individual transposon mutants were picked and grown overnight at 30°C with shaking at 150 rpm in 96-well plates containing 100 µl modified SM broth (recipe as described above, without agar, pH 6.0 to 6.4) per well. The following day, 5 µl from each well was plated on a corresponding 96-well modified SM agar plate, allowed to dry, and overlaid with 2 µl of *D. discoideum* at 500 cells/µl. Plates were monitored for up to 7 days for wells containing susceptible mutants, which were characterized by enhanced plaque formation and/or sporulation. Susceptible mutants were retested in replicates of 8 for confirmation of the phenotype (Table 2).

The location of the transposon insertion in each mutant was determined by inverse PCR (66). Briefly, DNA was extracted from each mutant using the E.Z.N.A bacterial DNA kit (Omega Bio-Tek, Norcross, GA) per the manufacturer’s directions. About 1 to 2 µg of genomic DNA was digested for 2 h at 37°C using HincII or PstI (New England Biolabs, Whitby, Ontario, Canada) in a 20-µl reaction volume using 20 units of enzyme and according to the manufacturer’s instructions. Ten microliters of heat-inactivated digest was added to 3 µl T4 DNA ligase (New England Biolabs), 167 µl double-distilled water (ddH_2_O), and 20 µl 10× T4 buffer to be incubated for 16 h at 16°C. Ligations were purified using the E.Z.N.A Cycle Pure kit (Omega Bio-Tek) and eluted into a 30-µl volume. Typically, 5 µl of purified ligation had sufficient template to be amplified in a 25- to 50-µl PCR mixture using EconoTaq DNA polymerase (Lucigen Corp., Middleton, WI) per the manufacturer’s instructions, using the primers NPT+772 (5′-TTCGCAGCGCATCGCCTTCTATC-3′) and NPT-41 (5′-AGCCGAATAGCCTCTCCACCCAAG-3′). Cycling conditions were one denaturation cycle of 94°C for 120 s, followed by 40 cycles of 94°C for 30 s, 63°C for 30 s, and 72°C for 240 s, and one polymerization cycle of 72°C for 300 s. Amplification was verified by gel electrophoresis, and single amplicons were excised and gel purified from any reactions with multiple amplicons using the E.Z.N.A gel extraction kit (Omega Bio-Tek) per the manufacturer’s instructions. Amplicons were sequenced by Génome Québec (Montréal, Québec, Canada). Insertion sites were confirmed by BLAST analysis against the annotated *P. ananatis* BRT175 genome (46).

### *rhlA-rhlB* mutant phenotypic characterization assays.

To determine the relative differences in grazing susceptibility caused by the interruption of *rhlA* and *rhlB*, cultures of *P. ananatis* BRT175, the *rhlA*::Tn*5* strain (mutant 28C8), and the *rhlB*::Tn*5* strain (mutant 32C5) were grown overnight at 30°C in modified SM broth. An 0.5-ml sample of each strain culture was added to 5 ml of fresh modified SM broth and incubated for another 40 min at 30°C to dilute the cultures and increase the proportion of live cells. Cell densities were standardized to an OD_600_ of ~0.125 from 200-µl samples using the plate spectrophotometer in 96-well plates (as described above). *D. discoideum* cells were diluted half-fold eight times starting at 1,600 cells/µl. A 12.5-µl volume of bacteria was spotted on modified SM agar and allowed to dry. Five microliters of each of the dilution series of *D. discoideum* cells was then placed on separate spots in order of decreasing concentration. Experiments were performed twice with two replicates each.

To create conditioned culture medium for the cytotoxicity assays, *P. ananatis* BRT175, *P. ananatis* BRT175 *rhlA*::Tn*5* (mutant 28C8), and *P. ananatis* BRT175 *rhlB*::Tn*5* (mutant 32C5) were each grown at 21°C for 6 days with shaking at 220 rpm in modified M9 medium (2 mM MgSO_4_, 20 g/liter glucose, 0.1 mM CaCl_2_, 1/5 dilution of 5× M9 salts [64 g/liter Na_2_HPO_4_, 15 g/liter KH_2_PO_4_, 2.5 g/liter NaCl, 13.78 g/liter NaNO_3_]). Sodium nitrate was substituted for ammonium chloride as it has been shown to be a preferred nitrogen source in *Pseudomonas aeruginosa* for surfactant production (67). Filtration was performed to retain products exported by the bacteria while excluding bacterial cells. Cultures were centrifuged, the bacterial pellet was discarded, and supernatants were filter sterilized using 0.2- to 0.22-µm polyether sulfone (PES) bottle top filters. The supernatants and control media were adjusted to pH 6.52 ± 0.02 and resterilized using 0.2- to 0.22-µm PES syringe filters to standardize pH values for downstream assays. Supernatants were kept at 4°C between experiments.

These supernatants were then used in PFU assays to assess *D. discoideum* cell viability after exposure to conditioned culture medium. Overnight cultures of *E. coli* B/r were centrifuged twice for 2 min at 12,000 × *g* and resuspended in 1 ml SorC each time. The final *E. coli* suspension was then diluted to an OD_600_ of ~0.1 (200 µl, 96-well plate). The *D. discoideum* cells (harvested as described above) were diluted to 15 cells/µl in SorC. Aliquots of 90 µl of modified M9 culture medium conditioned by *P. ananatis* BRT175, *P. ananatis* BRT175 *rhlA*::Tn*5* (mutant 28C8), or *P. ananatis* BRT175 *rhlB*::Tn*5* (mutant 32C5) or unconditioned culture medium as the control were prepared. Ten microliters of amoebae was then added to the 90 µl of medium and incubated for 25 min at 21°C. Three hundred microliters of the prepared *E. coli* suspension was added after incubation. The entire volume was pipetted on a 60-mm petri dish containing SM/5 medium and dried while on a rotary shaker to help evenly distribute the liquid. The plates were incubated at 21°C, and plaque counts were taken after 3 to 4 days. Statistical comparisons were performed using the Kruskal-Wallis test and Dunn’s test (68) for pairwise comparisons of the mean plaque counts from three independent experiments between the control and each treatment.

Cell viability was assessed using a trypan blue viability assay. This was performed by resuspending pelleted *D. discoideum* cells in 125 µl of filtered conditioned culture medium from either *P. ananatis* BRT175, *P. ananatis* BRT175 *rhlA*::Tn*5*, or *P. ananatis* BRT175 *rhlB*::Tn*5*, with unconditioned modified M9 medium as a control. A stock of *D. discoideum* cells was prepared at ~4,000 cells/µl for creating 250-µl aliquots for each replicate. The cells were centrifuged for 5 min at 500 × *g*, and the supernatant was discarded. *D. discoideum* cells were resuspended and exposed to the conditioned culture medium and control for a minimum of 20 min at room temperature. One hundred twenty-five microliters of 0.4% trypan blue was added, and total and unstained cells were enumerated by counting 25 of the 0.040-mm^2^ squares on a hemocytometer. Each treatment was performed in triplicate. Most cells in the wild-type-conditioned culture medium treatment could not be enumerated as they were reduced to cellular debris. Statistical comparisons were performed using the Kruskal-Wallis test and Dunn’s test (68) for pairwise comparisons of the mean viability counts from three independent experiments between the control and each treatment.

Swarming trials were performed by inoculating a single colony in the center of a modified SM agar plate containing 0.5% (wt/vol) agar and incubating it at 21°C for 24 h. *P. ananatis* BRT175 mutants were monitored for up to 48 h for swarming. Swarming assays were also performed on modified M9 agar plates containing 0.5% (wt/vol) agar by centrifuging cultures grown at 30°C and resuspending the bacterial pellet in the medium to an OD_600_ of ~0.4 (200 µl, 96-well plate). Ten microliters of concentrated culture was applied to the center of the agar plates and incubated at 21°C for 48 h. All images in this study were captured using an Epson Perfection V330 photo scanner at 600 to 1,200 dots per inch (DPI).

### Biosurfactant production and extraction.

To characterize the structure of the biosurfactant, solvent extractions were performed on culture medium conditioned with either *P. ananatis* BRT175 or *P. ananatis* BRT175 *rhlA*::Tn*5* (mutant 28C8). Cultures were grown in 200 ml MSM supplemented with 20 g/liter glucose and 2 g/liter NaNO_3_ in 1-liter Erlenmeyer flasks and incubated at 30°C with shaking at 250 rpm. MSM had the following composition: 2.5 g/liter KH_2_PO_4_, 1.5 g/liter K_2_HPO_4_, 0.1 g/liter CaCl_2_⋅2H_2_O, 0.4 g/liter MgSO_4_⋅7H_2_O, 2 ml/liter trace element solution (TES), pH adjusted to 7. The composition of TES was 2 g/liter FeSO_4_⋅7H_2_O, 1.5 g/liter MnSO_4_⋅H_2_O, 0.6 g/liter (NH_4_)_6_Mo_7_O_24_⋅4H_2_O, 1.4 g/liter ZnSO_4_⋅7H_2_O, 1.2 g/liter CoCl_2_⋅6H_2_O, 1.2 g/liter CuSO_4_⋅5H_2_O, and 2 g/liter sodium citrate⋅2H_2_O.

After 6 days of incubation, the cells were pelleted by centrifugation at 8,000 × *g* for 15 min, and the resulting supernatant was acidified with 1 N HCl to a pH of 2 to 3. The supernatant was then extracted twice with equal volumes of ethyl acetate. The organic fractions were then pooled and rotary evaporated, which yielded a crude extract containing the biosurfactant product. The final crude extract from 200 ml of culture was dissolved in 25 ml of MilliQ water to give an 8-fold-concentrated extract solution, which was subsequently used for further analysis.

### Liquid chromatography-mass spectrometry analyses.

The culture extracts of *P. ananatis* BRT175 and *P. ananatis* BRT175 *rhlA*::Tn*5* (mutant 28C8) were dissolved in methanol, and 16-hydroxyhexadecanoic acid was added as an internal standard. Samples were analyzed by high-performance liquid chromatography (HPLC; Waters 2795, Mississauga, Ontario, Canada) equipped with a 6.4- by 150-mm Agilent Zorbax Eclipse XDB-C_8_ reverse-phase column (particle size, 5 mm) using a water-acetonitrile gradient with a constant 2 mmol ⋅ liter^−1^ concentration of ammonium acetate (56). The detector was a mass spectrometer (Quattro Premier XE; Waters). Analyses were performed in negative electrospray ionization (ESI−), supplemented by the multiple-reaction monitoring (MRM) mode (69).

### Surface tension and emulsification assays.

The surface tension of the extract solutions of *P. ananatis* BRT175 and *P. ananatis* BRT175 *rhlA*::Tn*5* (mutant 28C8) strains was measured by the du Noüy ring method using a Fisher tensiometer model 20 (Fisher Scientific, Pittsburgh, PA). The instrument was calibrated against water, and measurements were performed in triplicate at room temperature. The emulsifying activity of culture extracts was tested against *n*-hexadecane. Three-milliliter aliquots of extract solutions of *P. ananatis* BRT175 and *P. ananatis* BRT175 *rhlA*::Tn*5* (mutant 28C8) were mixed with 2 ml of *n*-hexadecane and vortexed at high speed for 2 min. The emulsion was observed after letting the tubes stand at room temperature for 60 min. The lipophilic dye Sudan black was added to the *n*-hexadecane to increase contrast.

### Phylogenetic analyses.

Homologs of the *P. ananatis* BRT175 *rhlA* and *rhlB* genes were identified using BLAST against both complete and draft genome sequences available at NCBI. Alignments were made using ClustalX2 with default parameters using iteration after each alignment step. Neighbor-joining phylogenies were constructed using MEGA6 (70) with the Jones-Taylor-Thornton amino acid model, complete gap deletion, and 500 bootstrap replicates.

### Growth comparisons of *P. ananatis* BRT175 mutants.

*P. ananatis* BRT175 and Tn*5* mutants were inoculated into 10 ml of modified SM broth in a 15-ml conical tube, which was then tightly sealed and incubated overnight at 30°C without shaking (71). Overnight cultures were standardized to an OD_600_ of ~0.2 (300 µl, 96-well plate). Of the standardized culture, 50 µl was added to 250 µl of modified SM broth in a 96-well plate to be grown at 30°C for 18 h with continuous medium shaking, and OD_600_ readings were taken every 20 min in a BioTek Synergy HT plate reader. Each experiment was performed three times independently with 6 replicates per strain. Nine mutants were tested per plate, with *P. ananatis* BRT175 being used as a reference control for each set of mutants. Only one mutant per affected gene was tested (Table 2). Figure S1 in the supplemental material was generated in R using ggplot2 (72, 73).

### Nucleotide sequence accession numbers.

The *rhlA* and *rhlB* genes from *P. ananatis* 15320, BRT98, Cit30-11, 26SR6, and 17671 have been deposited in GenBank under accession numbers KM819089, KM819090, KM819091, KM819092, KT455465, KT455466, KT455467, KT455468, KT455469, and KT455470.
