# Supplementary figures and images for: A Novel Glycolipid Biosurfactant Confers Grazing Resistance upon Pantoea ananatis BRT175 against the Social Amoeba Dictyostelium discoideum
Source: mSphere. 2016 Jan 20;1(1):e00075-15. doi: 10.1128/mSphere.00075-15 (PMC4863597; doi:10.1128/mSphere.00075-15)

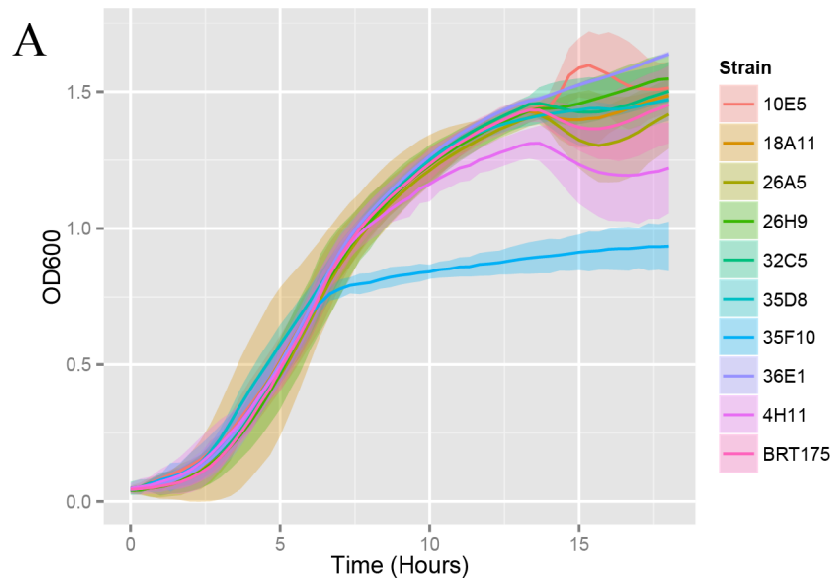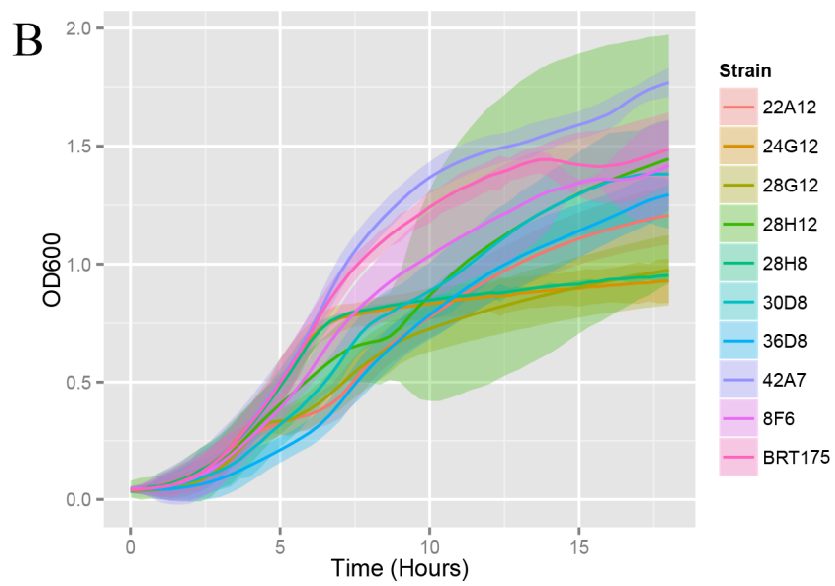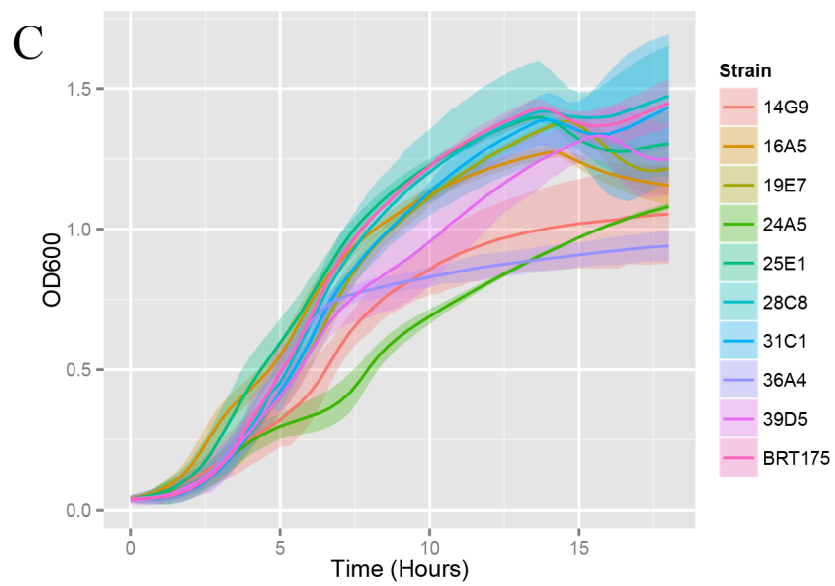

Supplement: Figure S1 [file sph001160041sf1.pdf]
